# Experimental Study on Dynamic Compressive Behaviors of Sand under Passive Confining Pressure

**DOI:** 10.3390/ma15134690

**Published:** 2022-07-04

**Authors:** Liang Li, Xinyu Kou, Gao Zhang, Kewei Huang

**Affiliations:** 1 Key Laboratory of Urban Security and Disaster Engineering, Beijing University of Technology, Ministry of Education, Beijing 100124, China; kouxy@emails.bjut.edu.cn (X.K.); zhanggao0529@163.com (G.Z.); 2 Fuzhou Academy of Building Research Co., Ltd., Fuzhou 350003, China; huangkewei12@163.com

**Keywords:** sand, dynamic compressive behaviors, passive confining pressure, impact dynamic response, particle breakage, SHPB

## Abstract

Dynamic compressive tests of sand under passive confining pressure were carried out using a Split Hopkinson Pressure Bar (SHPB) setup. The dynamic response, energy dissipation and particle-breaking behaviors of sand subjected to high-speed impact were investigated. Sand specimens with moisture contents of 0%, 2%, 4%, 8%, 10% and 12% and relative densities of 0.1, 0.5 and 0.9 were prepared. The variation in the strain rate was controlled between 90 s^−1^ and 500 s^−1^. The specimens were confined in a designed sleeve to create passive confining pressure. The experimental results show that the sand specimens were extremely sensitive to the strain rate. When the strain rate was less than 400 s^−1^, the stress and strain of the specimens increased with the increase in the strain rate but decreased when the strain rate exceeded 400 s^−1^. The peak strain and peak stress increased with the increase in the relative density. Particle breakage was aggravated with the strain-rate increase. Compared with the specimen without water, the relative breakage rate of the specimen with a moisture content of 12% decreased by 30.53% when the strain rate was about 95 s^−1^ and by 25.44% when the strain rate was about 460 s^−1^. The analysis of energy dissipation revealed the essential cause of sand destruction. The specific energy absorption rate increased with the increases in the initial relative density and moisture content.

## 1. Introduction

As a common geotechnical material, sand has been widely used as the foundation of superstructures or directly as a building material. It can be subjected to a variety of dynamic loads during its service life, such as mining, dynamic compaction [1], underground explosion [2] and shell impact. The description of the dynamic behaviors under high-strain-rate loading has a guiding effect for the design of civil buildings and military structures. However, sand is a typical loose material with strong regional characteristics. The physical properties of sand in different environments, such as moisture content, density, etc., have certain differences [3,4,5,6]. The differences further aggravate the challenges in assessing the response of sand subjected to high-speed impact, including dynamic deformation.

Sand deformation is caused by particle breakage, which is a manifestation of energy dissipation. The shape and size of particles [7,8], saturation with water [9] and particle gradation [10,11] have a significant impact on the macro compression mechanism [12,13,14]. Particle shape and size determine the phenomenon and degree of sand damage [15]. Circular sand particles are less likely to break than irregular sand particles due to their lower stress concentration [16]. The probability of breaking small particles is smaller than that of breaking large particles, because the breaking of small particles requires more energy. The compression rate of sand is sensitive to the saturation rate. Zhao et al. [17] confirmed that unsaturated sand compressed faster and was more prone to creep than dry sand. The grain-size distribution has a great influence on the compression of sand. Sand with good gradation has poor compressibility [18], mainly due to the fewer pores in the sand preventing sand particles from slipping [19].

The Split Hopkinson Pressure Bar (SHPB) is widely used for researching the dynamic mechanical properties of various geotechnical materials subjected to high-speed impact [20], such as frozen soil [21,22], clay [23,24,25] and sand [26]. The compressive strength of frozen soil subjected to high-speed impact is sensitive to the strain rate and temperature [27,28]. Clay exhibits a rebound phenomenon under passive confining pressure, and the degree of rebound increases with the increase in the strain rate. The peak stress and strain of clay increase significantly with the increase in the strain rate [29].

However, the dynamic behavior of sand subjected to high-speed impact is different. Sand is a kind of low-impedance bulk material [30]; the low wave velocity and high wave attenuation rate in sand specimens are caused by the large amount of void air in sand [31]. It is difficult to ensure one-dimensional stress balance during an SHPB impact test. The waveform shaper is used as a common solution to increase the amplitude and duration of the incident wave. The impact of the bullet on the bar is cushioned, and the signal is stabilized. The recorded waveform is smoother. Parameters such as amplitude, wavelength and incident time can be easily determined. The waveforms of different materials have been used by researchers [9,32]. Song et al. [33] compared the test results of wave shapers with different materials (copper plate, paper and thin rubber) and found that thin rubber owned the best shaping effect. Besides promoting the dynamic stress balance of the material, the employment of a well-designed waveform shaper enables one to reduce the dispersion pulse and constant strain rate [34].

At present, many researchers have investigated the dynamic mechanical properties of dry sand and unsaturated sand. The mechanic behavior of dry sand after dynamic compression is highly sensitive to the initial mass density [35]. Luo et al. [36] indicated that dry sand with high mass density had poor dynamic compressibility. The higher the initial mass density of dry sand is, the lower the initial porosity is. The initial porosity of dry sand can offset deformation when subjected to dynamic compression. On the contrary, the stress and strain of dry sand with high initial porosity can be enhanced under high-strain-rate compression [37]. The dynamic mechanical properties of unsaturated sand are affected by the moisture content [38]. The stiffness of unsaturated sand decreases slightly with the increase in the moisture content. However, Ross et al. [39] believed that the moisture content affects the effective stress, which means that the moisture content of sand subjected to high-speed impact has a large effect on the stiffness of sand. In their study, with the increase in the moisture content, the stiffness of sand first increased and then decreased, and sand was the hardest when the moisture content was 34.8%. When the saturation was beyond 40%, the compressive strength of sand was obviously improved, and the specimen with 20% saturation was more difficult to compress than dry sand [40]. Cai et al. [41] proved that the moisture content had no significant effects on the p-wave velocity, but transmittance showed an upward trend with the increase in the moisture content. However, the above studies are based on the influence of compactness and moisture content at a certain strain rate. It is still questionable whether these effects have a consistent trend at different strain rates.

In addition, the discussion of the energy dissipation of sand under high-strain-rate loading has a great significance in applications related to civil buildings and military structures. However, the existing research studies exploring the energy of sand subjected to dynamic impact are very limited. Ou Yang et al. [42] found that the energy absorption efficiency of sand was insensitive to the change in the water content when subjected to a single impact but decreased with the increase in the relative density. When subjected to repeated impacts, the influence of the moisture content and relative compactness on the energy absorption efficiency of sand was further reduced. Wang et al. [30] concluded that when the water content was 0–50%, the energy absorption of sand decreased with the increases in the initial density and lateral constraint. However, the combined effect of water content and different strain rates on the energy dissipation of sand is not clear. Further studies need to be carried out to systematically analyze the effects of changes in the moisture content, strain rate and relative density on the energy absorption of sand subjected to impact.

In the current study, a series of dynamic compression tests were conducted to investigate the dynamic behaviors of sand. Fujian standard sand with particle sizes of 0.25–1 mm was selected. The SHPB was modified to ensure the stress balance state of the sand specimens during dynamic impact. A confining steel sleeve was designed to connect with the SHPB and to confine the sand specimens subjected to impact loads. The failure phenomena of sand specimens subjected to a dynamic impact load were analyzed. The influence of the strain rate, relative density and moisture content on dynamic compression mechanical properties was examined. The degree of sand particle breakage was further quantitatively evaluated considering the relative breakage rate. The essential characteristics of sand particle breakage were studied by analyzing energy dissipation.

## 2. Experimental Setup and Material

### 2.1. Material Properties

**Figure 1:**
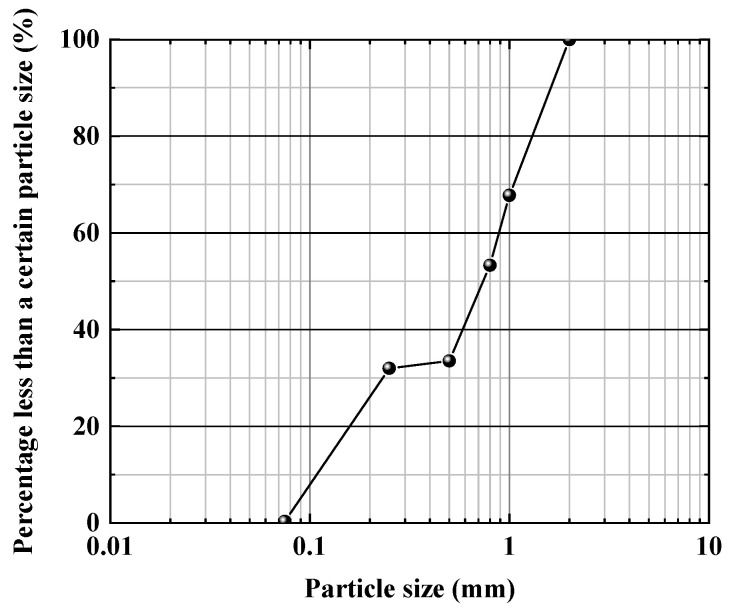
Particle gradation curve of the original sand specimen.

**Figure 2:**
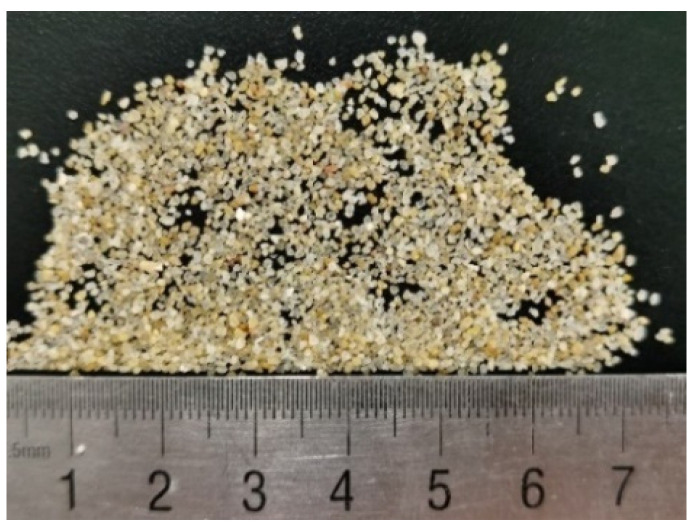
Tested sand specimen after screening.

The sand selected for the current research study was Fujian standard sand. The main component of the tested sand was SiO_2_, with a content of more than 96%. Therefore, it could be referred as siliceous sand. The original grain-size distribution curve is given in Figure 1. The test sand was screened as medium-coarse sand with particle sizes of 0.25–1 mm (Figure 2). The physical parameters of the selected sand are summarized in Table 1. According to the expression of the density state of non-cohesive soil with relative density $D_{r}$, the relative density of the test sand could be defined as:

(1) $$D_{r}=\frac{\rho_{\mathrm{dmax}}\left(\rho_{d}-\rho_{\mathrm{dmin}}\right)}{\rho_{d}\left(\rho_{\mathrm{dmax}}-\rho_{\mathrm{dmin}}\right)}$$

where $\rho_{d}$, $\rho_{\mathrm{dmax}}$ and $\rho_{\mathrm{dmin}}$ are the initial, maximum and minimum dry densities. The value of $D_{r}$ was between 0 and 1.

### 2.2. Experimental Setup

**Figure 3:**
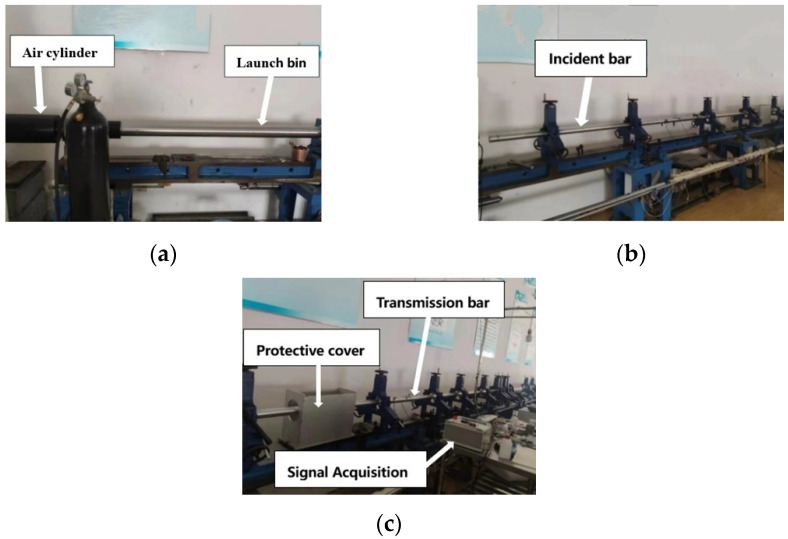
SHPB setup used in current test study. (**a**) Air cylinder and launch bin; (**b**) Incident bar; (**c**) Transmission bar, protective cover and signal acquisition.

**Figure 4:**
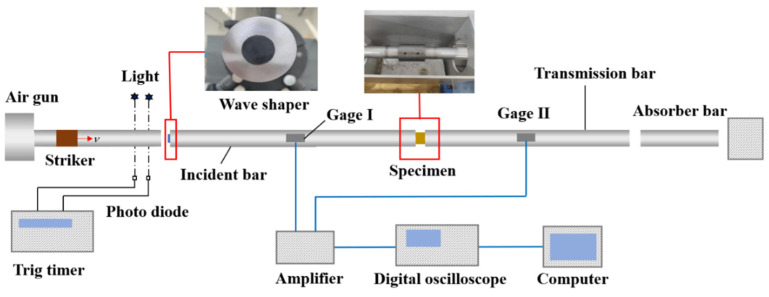
System composition of SHPB test setup.

The dynamic compressive experiments were carried out using the SHPB setup obtained from Hefei JiangShui Dynamic Mechanics Experimental Technology Co. Ltd. (Hefei, China). The SHPB test setup used in the current test study is shown in Figure 3. The classical SHPB consisted of impact bullet, incident bar, transmission bar, velocity detector and stress–strain variable measurement system. The system composition of the SHPB test setup is presented in Figure 4. The incident bar and transmission bar had a diameter of 50 mm and were made of LV4 aluminum alloy. The lengths of the incident bar and transmission bar were 4000 mm and 3250 mm, respectively. The density of the bar was 2700 kg/m^3^, and the wave speed was 5090 m/s. The material and diameter of the impact bullet were consistent with the incident bar; the length was 400 mm, and the mass was about 8.5 kg.

**Figure 5:**
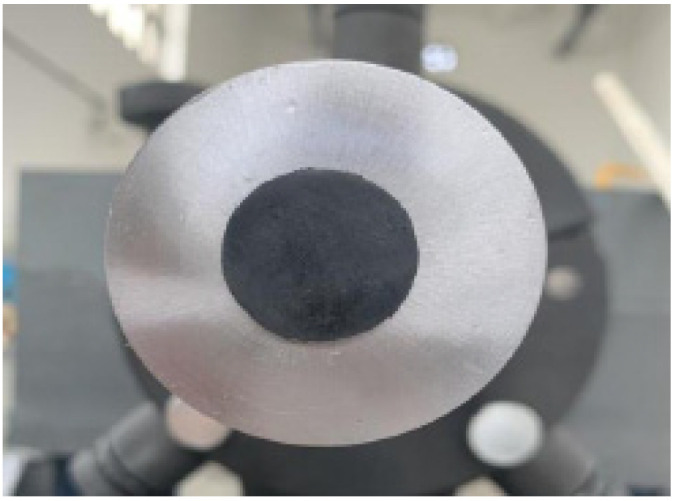
Waveform shaper.

The SHPB was calibrated to ensure the accuracy and effectiveness of the test results. A 7.5 mm diameter rubber waveform shaper was sticked on the impact end of the incident bar, as presented in Figure 5. The stress balance state of the specimens after using the shaper is discussed in Chapter 3. Pre-tightening force was applied to the specimens by tightening the rubber belt tied to the incident bar and the transmission bar. This step could ensure the close contact between the specimens and the incident bar or the transmission bar. Moreover, the energy diffusion of the contact surface was prevented during loading.

The bullet was driven by compressed air to impact the incident bar to complete the dynamic compression loading of a specimen. The incident wave was formed in the incident bar. The signal could be measured by the strain gauge on the incident bar. The incident wave propagated to the specimen. Due to the wave impedance inconsistency between the specimen and the bar, part of the wave was reflected, and the other part was transmitted to the transmission bar through the specimen. The signals of the two waves could be measured by the strain gauge on the bar. Different impact velocities could be obtained by applying different air pressures. The impact velocity could be recorded by a tachometer. Base on the assumption of the one-dimensional stress wave and the uniformity of the specimens, the relationships among the incident wave, the reflected wave and the transmitted wave could be used to obtain the stress–strain relationship equations of the specimens under dynamic compression:

(2) $$\sigma_{s}\left(t\right)=\frac{EA}{A_{s}}\varepsilon_{t}\left(X_{G2},t\right)=\frac{EA}{A_{s}}\left[\varepsilon_{i}\left(X_{G1},t\right)+\varepsilon_{r}\left(X_{G1},t\right)\right]$$

(3) $${\dot{\varepsilon }}_{s}\left(t\right)=-\frac{2c_{0}}{l_{s}}\varepsilon_{r}\left(X_{G1},t\right)=\frac{2c_{0}}{l_{s}}\left[\varepsilon_{i}\left(X_{G1},t\right)-\varepsilon_{t}\left(X_{G2},t\right)\right]$$

(4) $$\varepsilon_{s}\left(t\right)=-\frac{2c_{0}}{l_{s}}\int_{0}^{t}\varepsilon_{r}\left(X_{G2},t\right)dt=\frac{2c_{0}}{l_{s}}\int_{0}^{t}\left[\varepsilon_{i}\left(X_{G1},t\right)-\varepsilon_{t}\left(X_{G2},t\right)\right]dt$$

where $\sigma_{s}$ and $\varepsilon_{s}$ are the stress and strain of the test specimen, respectively; ${\dot{\varepsilon }}_{s}$ is the strain rate of the test specimen; A and $A_{s}$ are the cross-sectional area of the pressure rod and the cross-sectional area of the test specimen, respectively; $l_{s}$ is the length of the test specimen; $\varepsilon_{i}\left(X,t\right)$, $\varepsilon_{r}\left(X,t\right)$ and $\varepsilon_{t}\left(X,t\right)$ are the incident, reflected and transmitted stress waves measured by the strain gauges, respectively; X is the position; t is the time; subscripts $G_{1}$ and $G_{2}$ of X are the numbers of the strain gauge; E is the Young’s modulus; and $c_{0}$ is the wave speed.

### 2.3. Specimen Preparation and Installation

**Figure 6:**
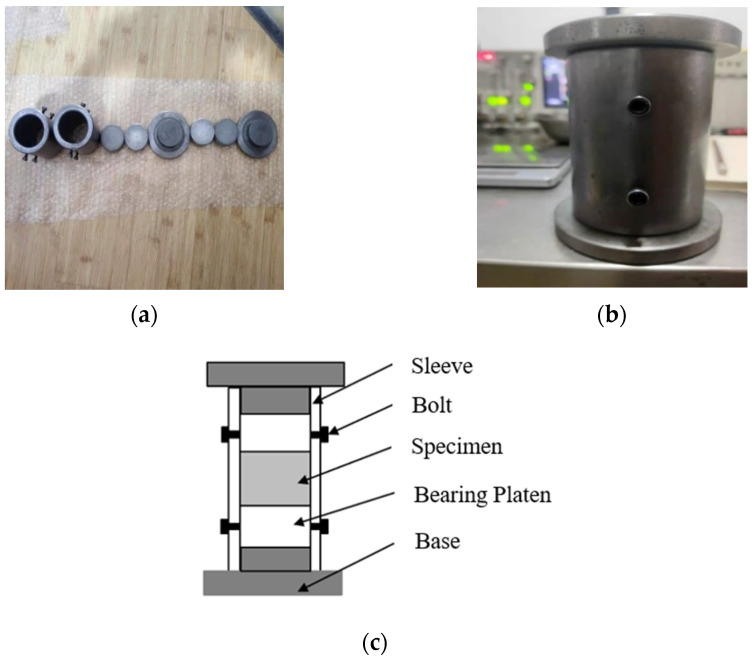
Sleeve, bearing platen and base for specimen fabrication. (**a**) Sleeve, bolts, bearing platens and bases.; (**b**) Assembled steel sleeve; (**c**) Specimen installation diagram.

The sand specimens were confined with the steel sleeve shown in Figure 6. The steel sleeve was combined with a pair of bearing platens and bases for specimen fabrication. When a sand specimen was subjected to axial dynamic compression, its radial direction tended to expand. The specimens were confined under passive confining pressure due to the constraint of the steel sleeve. A steel sleeve with an inner diameter of 50.05 mm was used in the current tests. A pair of LV4 aluminum alloy cylindrical platens with a diameter of 50 mm and a pair of steel bearing platens and bases were used to control the thickness of the specimens. Four bolts were set on the sleeve for fixing, which were made of M5 aluminum alloy. The inner wall of the sleeve and the side wall of the platen were polished smoothly to reduce friction.

The SHPB test results are sensitive to the inertial effects of the specimens in the axial and radial directions. The ratio of length to diameter of a specimen is defined as the aspect ratio. A specimen aspect ratio between 0.4 and 0.6 can significantly improve the accuracy of the test results [43]. Song et al. [33] analyzed the influence factors of the stress balance of soft materials and found that with specimens with lower thickness, it was easier to achieve stress balance. In this study, the diameter of the specimens matched the diameter of the incident bar. The aspect ratio of the specimens was 0.5. The length of the specimens was 25 mm. After the volume was selected, sand specimens with different relative densities could be obtained with different sand mass values. Different moisture contents by mass could be obtained by adding different values of mass of water to the specimen. It is noted that sand had to be fully dried before specimens with different moisture contents could be configured. The prepared specimens were sealed with plastic wrap to prevent moisture evaporation.

**Figure 7:**
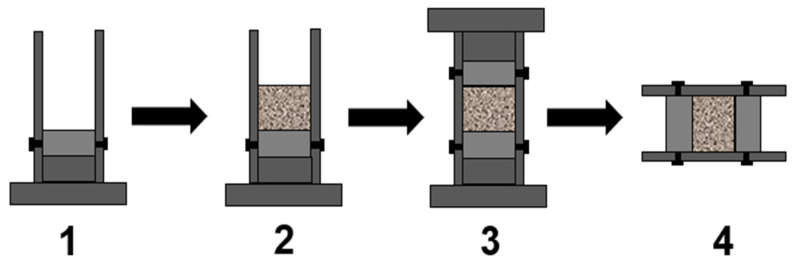
Steps of specimen fabrication. 1. Install the bottom base; 2. Install the sand; 3. Install the top base; 4. Placing horizontally.

**Figure 8:**
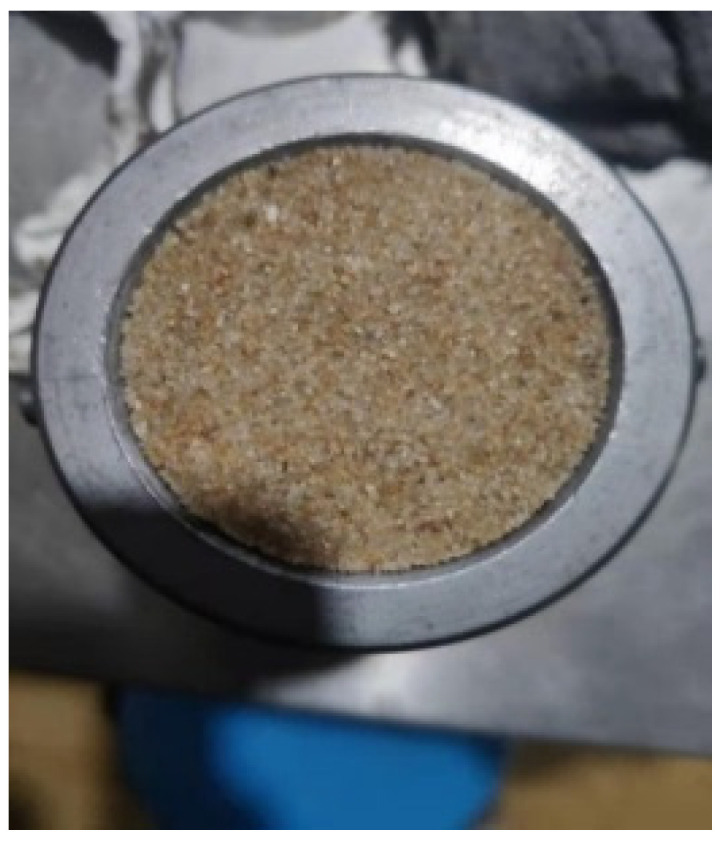
Fabricated sand specimen.

**Figure 9:**
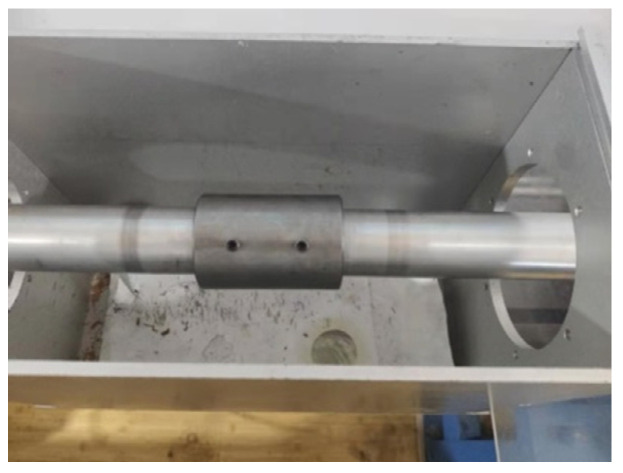
Specimen installation.

The fabrication steps of the specimens are shown in Figure 7. Firstly, the tube was installed on the bottom base, and the bottom spacer was inserted. Secondly, a sand specimen was put into the sleeve. In order to ensure the uniformity, the sand specimen was divided into three parts of equal mass and put into the steel sleeve in layers. The surface of each layer was smoothed with the smoother. The specimen was compacted by the compactor according to a predetermined compaction frequency. Thirdly, the top platen was installed, and the top base was placed. The top base was hammered with the compactor so that it was in close contact with the platen to obtain a constant thickness of the specimen. Then, the bolts were tightened to fix the sleeve, and the base was removed. The steel sleeve with the specimen was placed horizontally for a period of time. Finally, Vaseline was applied to the surfaces of both pads to reduce friction. The sleeve was sandwiched between the transmission bar and the incident bar. One of the fabricated sand specimens is shown in Figure 8, and specimen installation is illustrated in Figure 9.

### 2.4. Loading Cases

The strain rate effects of the dynamic response of specimens were studied by dynamic compressive loading at different impact velocities. The effects of the moisture content and relative density on the dynamic response characteristics of specimens were researched. The loading cases of the current test study are listed in Table 2. A total of 45 test conditions were set with different parameter combinations. In order to ensure the accuracy of the test, the test of each loading case was repeated three times. The optimal value, which was the test result closest to the average value, was taken as the test result. The strain rates of the sand specimens corresponding to impact velocities of 6 m/s, 7 m/s, 9 m/s, 11 m/s and 12.5 m/s were 90–100 s^−1^, 184–200 s^−1^, 275–300 s^−1^, 375–385 s^−1^ and 454–465 s^−1^, respectively.

## 3. Results and Discussion

**Figure 10:**
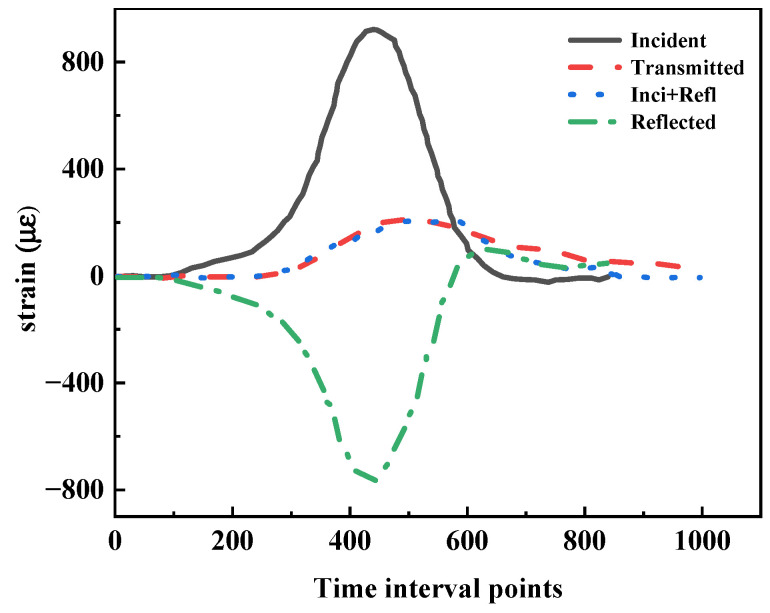
Typical incident, reflected and transmitted pulses in the stress balance test.

The stress balance of specimens is an important basis to verify the validity of SHPB test results. The stress balance of a specimen can be expressed as:

(5) $$\varepsilon_{1}=\varepsilon_{2}+\varepsilon_{3}$$

where $\varepsilon_{1}$, $\varepsilon_{2}$ and $\varepsilon_{3}$ are the strains corresponding to the incident, transmitted and reflected signals, respectively. Let $t_{r}$ be defined as the starting point of the reflected wave and $t_{t}$ be defined as the starting point of the transmitted wave; we can obtain:

(6) $$t_{r}=t_{0}+\frac{2l_{1}}{c}$$

(7) $$t_{t}=t_{0}+\frac{l_{1}+l_{2}}{c}+\frac{l_{s}}{c_{s}}$$

where $l_{1}$ is the distance between the strain gauge on the incident bar and the specimen; $l_{2}$ is the distance between the strain gauge on the transmission bar and the specimen; $l_{s}$ is the length of the specimen; c and $c_{s}$ are the wave velocities in the bar and in the specimen, respectively; $t_{0}$ is the incident wave head. The stress wave transmission in a dense specimen is efficient under the condition of lateral deformation constraint [44]. This means that with dense specimens it is easier to reach stress balance. The current study verified the stress balance of loose specimens with *D*_r_ = 0.1. The results are presented in Figure 10. It could be found that the time-dependent strains on both sides of a specimen were matched before the peak value was reached during impact. The specimens were in a state of dynamic stress balance. The aspect ratio of the specimens selected in Chapter 2.3 was reasonable. The particles of the specimens were broken, and the internal structure was reconstructed after impact. It was difficult for the specimens to reach stress balance, which was consistent with the test results. The loading path of the specimens is illustrated in Figure 10. It included a loading process and an unloading process. The stress of the specimens increased from the initial zero value to the peak value during the loading process and decreased from the peak value to the ultimate zero value during the unloading process.

**Figure 11:**
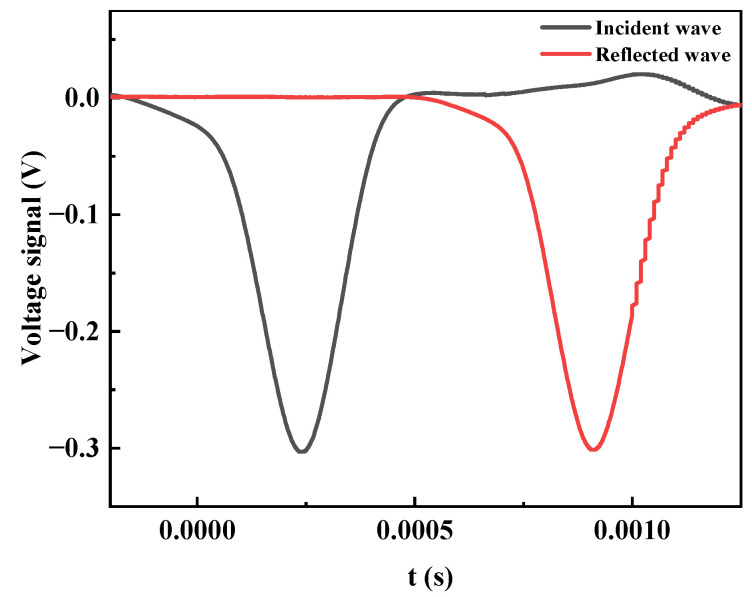
Recorded signal for impact with no specimens.

In the current test study, a steel sleeve was used to impose passive confining pressure on the specimen. In order to determine the effects of the sleeve and platen on the deviation of the test results, SHPB tests without sand specimens were conducted. It should be noted that the lubrication of the sleeve needed to be performed even though no specimens were installed. The transmission of the incident wave was observed when only the sleeve and platen were installed for impact. The result is given in Figure 11. The peak voltage of the incident wave and the transmitted wave were −0.305 V and −0.289 V, respectively. The peak value of the reflected wave was 0.014 V. Substantially, all the incident waves were transmitted into the transmission bar through the steel sleeve and platen. It can be considered that the sleeve and platen had no influence on the dynamic compression test results of the sand specimens.

The sand specimens were compressed, and their deformation was different after the impact test. In general, the deformation was unrecoverable plastic deformation. Moreover, the sand specimens were softened or hardened due to different initial relative density and moisture content, which is discussed in detail in this section.

### 3.1. Particle Breakage Analysis

In order to compare the crushing degree of sand particles after dynamic impact, sand specimens with initial relative density of 0.9 and initial moisture contents of 0%, 4%, 8% and 12% after loading were selected for analysis. The above sand specimens were subjected to a single impact at bullet velocities of 6 m/s, 7 m/s, 9 m/s, 11 m/s and 12.5 m/s.

**Figure 12:**
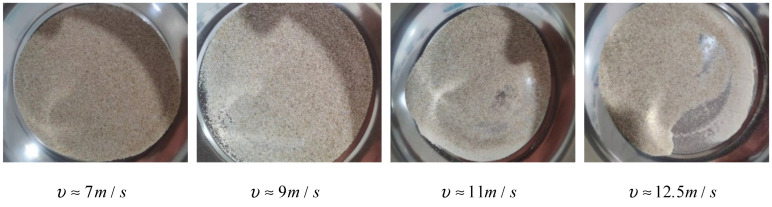
Failure phenomena of the dry sand specimens with initial relative density of 0.9 after impact loading.

The failure phenomena of the sand specimens after dynamic compression tests at different strain rates are presented in Figure 12. It can be clearly observed that the extent of sand particle breakage was aggravated as the strain rate increased. The change in the macroscopic mechanical properties of the materials was due to the slip, fragmentation and recombination of microscopic grains. Grain-size distribution curves can be used to quantitatively evaluate the degree of particle breakage, which is also related to the definition of the particle breakage coefficient or index [45,46]. It can be understood that the particle size and distribution of sand are important parameters affecting the mechanical properties of materials [34].

**Figure 13:**
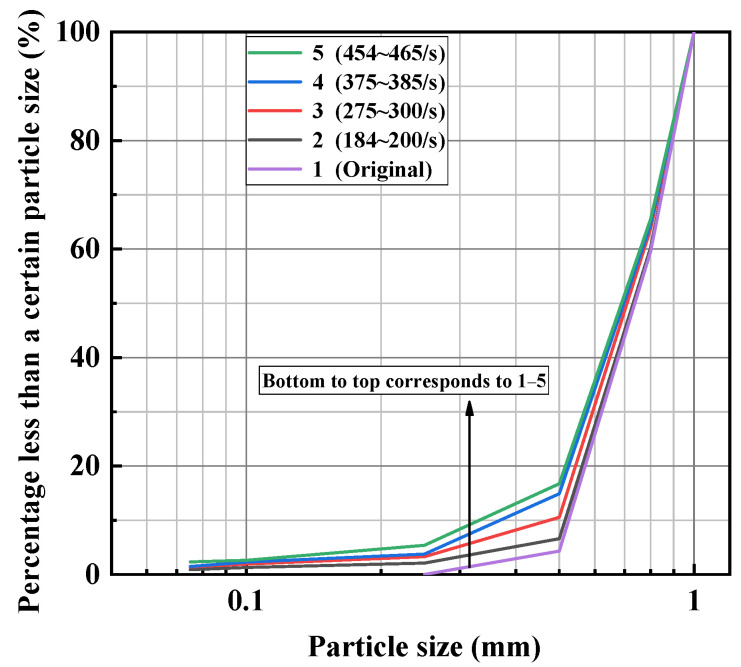
Grain distribution curves of sand specimens after impact loading.

The grain-size distribution curves of broken sand specimens were obtained via screening tests. The grain-size distribution curves of the sand specimens before and after dynamic compression are presented in Figure 13. The curves gradually move to the left and up with the increase in the strain rate. It was found that the increase in the strain rate led to a better gradation. With the increase in the strain rate, more large sand particles broke up into smaller ones. The range of particle sizes was enlarged, and the proportion of small particles increased. The pores among the large particles could be filled by the small particles. This corresponded to a better particle gradation.

#### 3.1.1. Effect of Strain Rate

The effect of the strain rate on particle breakage can be expressed by the relative breakage rate. Using the relative breakage rate, $B_{r}$ [45], we could quantify the amount of particle crushing in the current research study. Hardin [47] proposed that soil particles smaller than 0.074 mm could only be broken under extremely high stress. Therefore, 0.074 mm was adopted as the boundary size of particle breakage. Based on the initial grading curve, $B_{r}$ can be defined as:

(8) $$B_{r}=\frac{B_{t}}{B_{p}}$$

where $B_{p}$ is the breakage potential, defined as the area enclosed by the initial grain-size distribution curve and the vertical line segment with a particle size of 0.074 mm. $B_{t}$ is defined as the area enclosed by the grain-size distribution curve before and after breaking and the vertical line segment with a particle size of 0.074 mm. The breakage potential is defined as:

(9) $$B_{p}=\int_{0}^{1}b_{p}df$$

where $b_{p}$ is the particle-breakage-potential density function and f is the corresponding particle-size screening qualification rate. When d is expressed as the particle size of sand particles, $b_{p}$ is defined as:

(10) $$b_{p}=\left\{ \begin{array}{l} {log}_{10}\left(d/0.074\right)\\ 0 \end{array}\right.\begin{array}{l} ,d\geq 0.074\mathrm{mm}\\ ,d<0.074\mathrm{mm} \end{array}$$

The current crushing potential can be defined as:

(11) $$B_{t}=\int_{0}^{1}\left(b_{\mathrm{pb}}-b_{\mathrm{pa}}\right)df$$

where $b_{\mathrm{pb}}$ is the quantity of $b_{p}$ before the sand particles break and $b_{\mathrm{pa}}$ is the quantity after the sand particles have broken.

**Figure 14:**
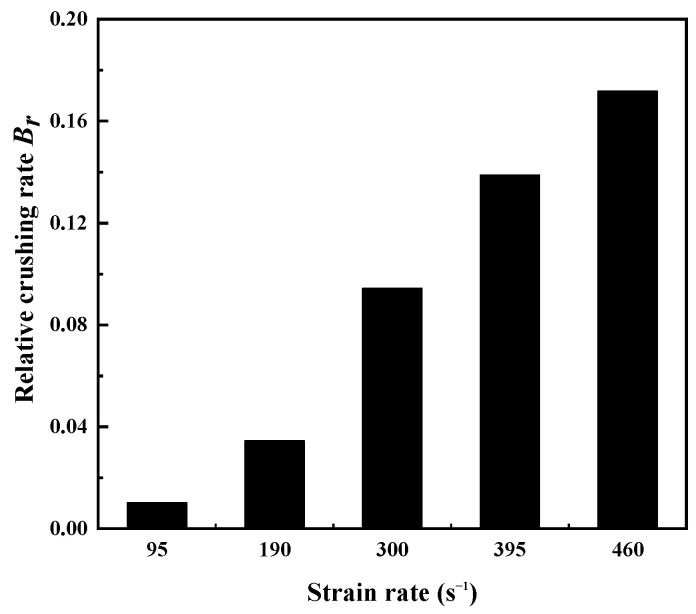
Relative crushing rate of dry sand specimens with initial relative density of 0.9 for different strain rates.

The influence of the strain rate on $B_{r}$ is shown in Figure 14. It was found that $B_{r}$ increased with the increase in the strain rate, and the growth rate gradually slowed down. The particle breakage of sand tended to be stable. Sand had a failure limit when subjected to dynamic impact. This may have been due to the fact that particle breakage is a process of energy dissipation and transfer [48]. A large amount of energy was input into the specimens during high-speed impact, which further increased the sand particle breakage rate. On the other hand, the steel sleeve, which made lateral expansion difficult, was used to restrain the specimens in the test. This prevented the mobilization and rearrangement of particles [30]. These two factors double-aggravated the particle breakage rate.

#### 3.1.2. Effect of Moisture Content

**Figure 15:**
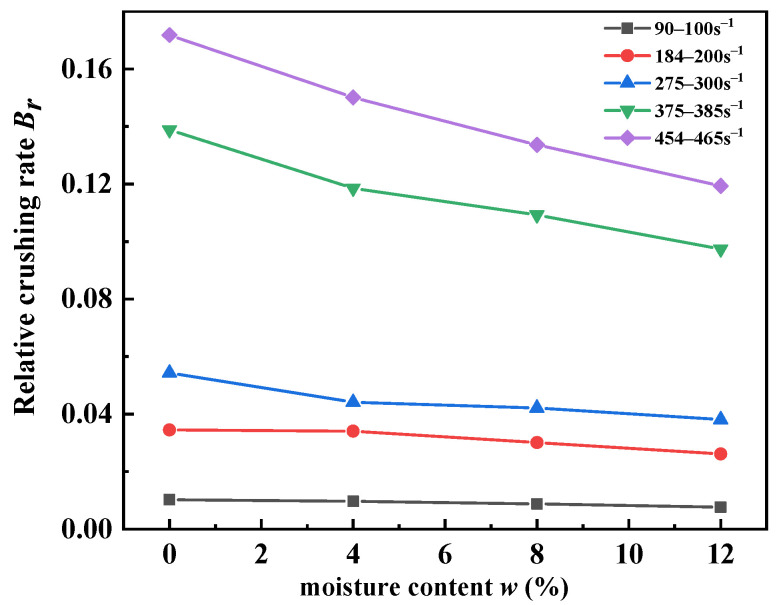
Relative crushing rate of sand specimens for different moisture contents.

Figure 15 indicates the influence of moisture content on $B_{r}$. It can be noticed that $B_{r}$ decreased with the moisture content, and the reduction rate increased with the increase in the strain rate. Water molecules were present in the pores of the unsaturated sandy soils. Pore water suffered some of the stress during the dynamic impact test. The effective stress of the sand particles decreased, and the particle breakage rate decreased. This may have been due to the protective effect of pore water on sand particles. The lubrication of pore water provided a suitable environment for the rearrangement of sand particles, which meant that less sand particles were broken. This lubrication also occurred between the steel sleeve and the specimens. It is evident that the high-speed impact amplified this lubrication.

### 3.2. Dynamic Response Behaviors of Sand

#### 3.2.1. Effect of Strain Rate

**Figure 16:**
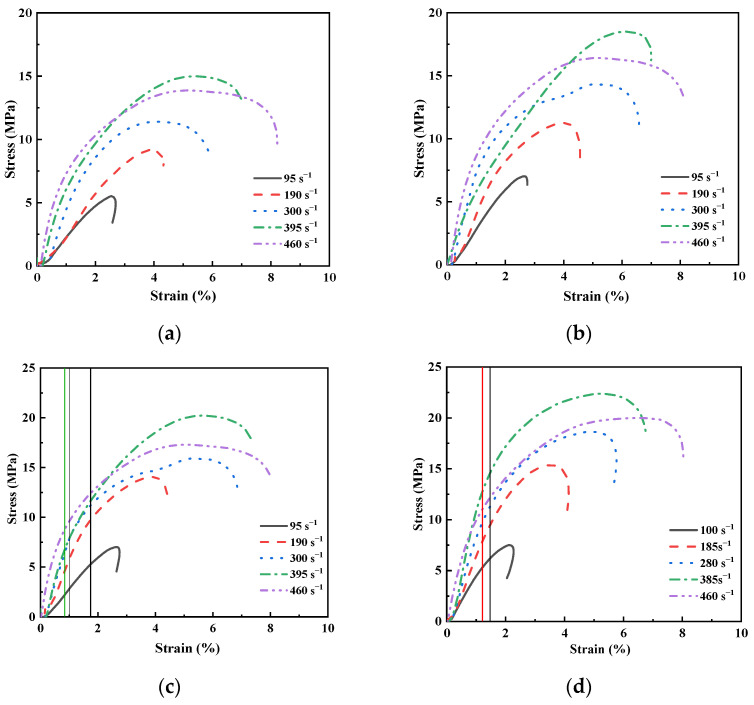

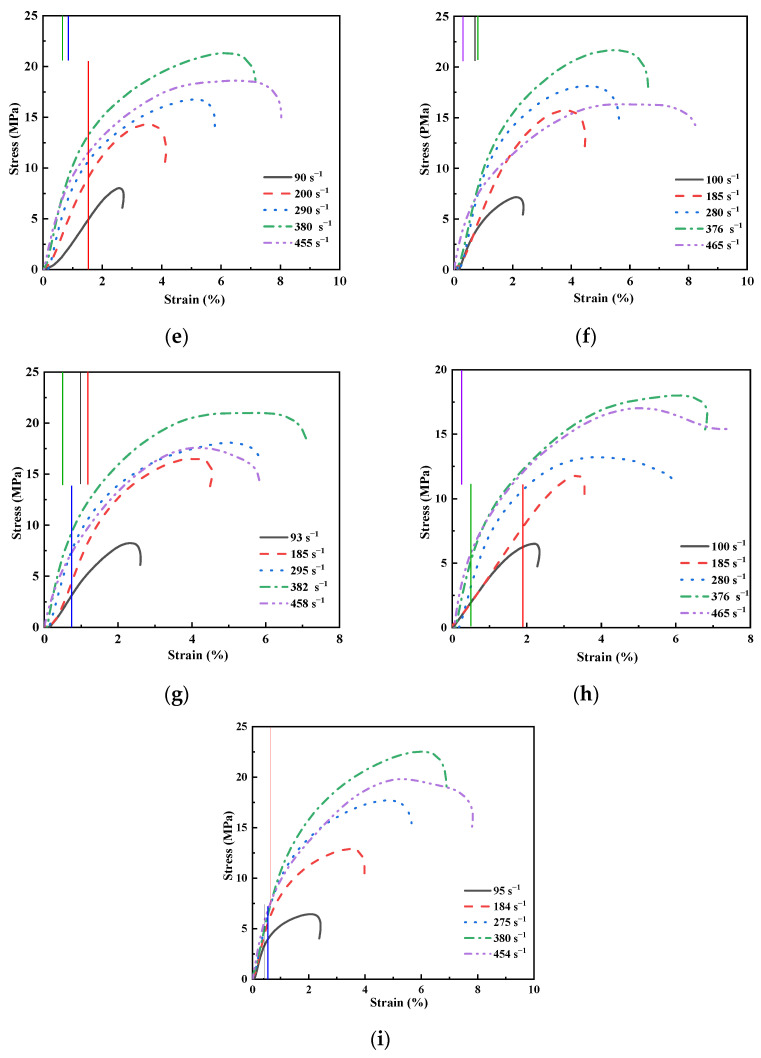
Dynamic stress–strain curves of sand specimens at different strain rates. The color of the vertical line corresponds to that of the strain rate. (**a**) w=0, $D_{r}=0.1$. (**b**) w=0, $D_{r}=0.5$. (**c**) w=0, $D_{r}=0.9$. (**d**) w=2%, $D_{r}=0.9$. (**e**) w=4%, $D_{r}=0.9$. (**f**) w=6%, $D_{r}=0.9$. (**g**) w=8%, $D_{r}=0.9$. (**h**) w=10%, $D_{r}=0.9$. (**i**) w=12%, $D_{r}=0.9$.

The dynamic stress–strain curves for sand specimens with different moisture contents and relative densities at different strain rates are presented in Figure 16. It is evident that the analyzed sand showed significant strain-rate dependency under dynamic compression. The strain rate had an enhancing effect on the dynamic stress and strain within a strain rate range from 100 s^−1^ and 400 s^−1^ but had a weakening effect when the strain rate was increased to 455 s^−1^. The vertical line in Figure 16 is the boundary between the elastic and inelastic phases of the stress–strain curve. In general, sand entered inelastic deformation faster with the increase in the strain rate.

The rate of particle breakage increased with the increase in the strain rate. The high-speed impact caused the fraction of large grains to become smaller, and a lot of small, tiny particles were produced. The pores among the sand particles were filled, resulting in a decrease in the deformation capacity of sand. The deformation ability of sand under dynamic compression was limited and was about 6%. The sand particles near the end of the incident bar were rapidly broken when the strain rate reached 455 s^−1^. The sand specimens first suffered local failure, resulting in a reduction in the peak stress.

#### 3.2.2. Effect of Initial Relative Density

**Figure 17:**
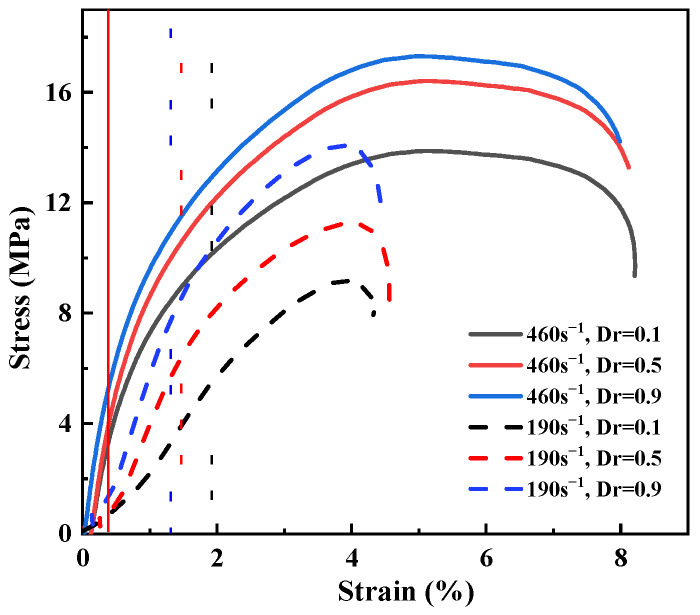
Dynamic stress–strain curves of sand specimens with different relative densities.

**Figure 18:**
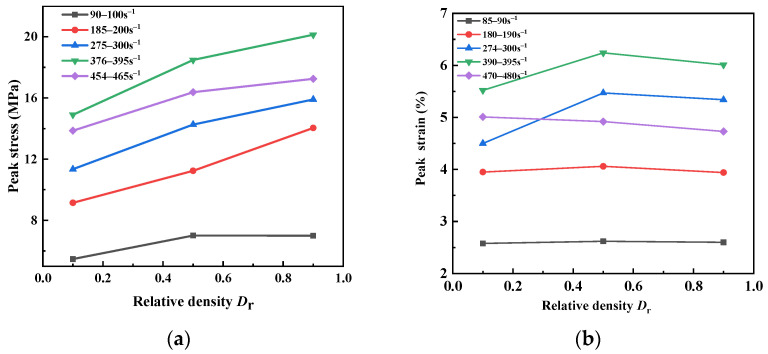
Peak stress and peak strain with relative density for sand specimens at different strain rates. (**a**) Peak stress; (**b**) Peak strain.

The dynamic stress–strain curves of sand specimens with different relative densities at 460 s^−1^ and 190 s^−1^ are presented in the Figure 17. It is obvious that sand specimens showed a significant density effect at both high strain rates and low strain rates. The stiffness of the specimens increased with the increase in the initial relative density, which means that the specimens became harder. Sand entered the inelastic stage faster as the relative density increased. The variations in the peak stress and peak strain with relative density for sand specimens at different strain rates are given in Figure 18. When the strain rates were 90~100 s^−1^, 184~200 s^−1^, 275~300 s^−1^, 376~395 s^−1^ and 454~465 s^−1^, the peak stress of the dense specimen (*D*_r_ = 0.9) increased by 28.0%, 53.4%, 28.7%, 35.1% and 24.5%, respectively, compared with the loose specimen (*D*_r_ = 0.1). The peak strain of the specimens tended to be stable after rising. The pores inside the sand became smaller, as the sand was denser. The friction and biting force among sand particles were enhanced, which improved the strength of the sand particles. When the strain rate exceeded a certain value, particle breakage occurred more frequently with the closer contact among sand particles. The broken particles filled the internal pores of the specimen, and the deformation capacity of the specimens tended to be stable.

#### 3.2.3. Effect of Moisture Content

**Figure 19:**
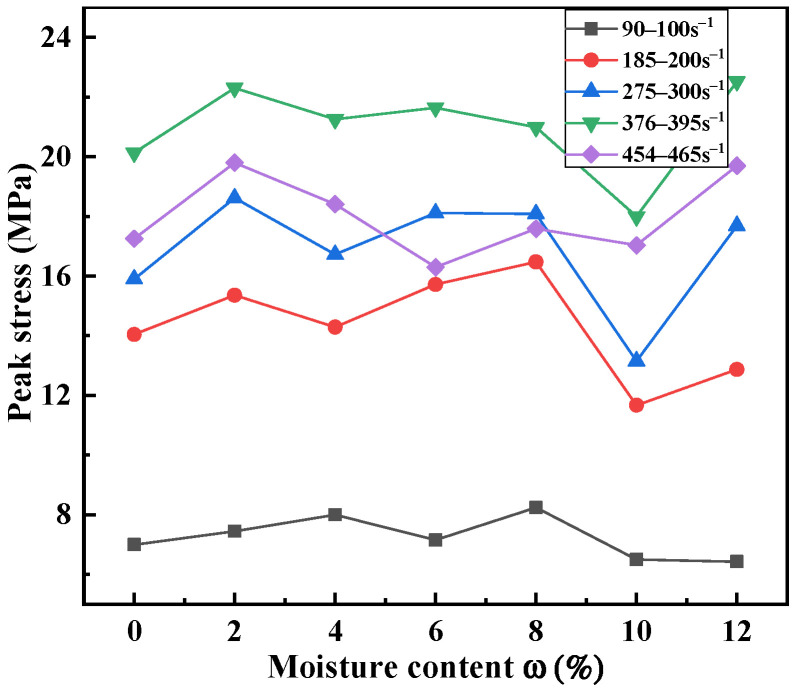
Variations in peak stress with moisture content for sand specimens at different strain rates.

**Figure 20:**
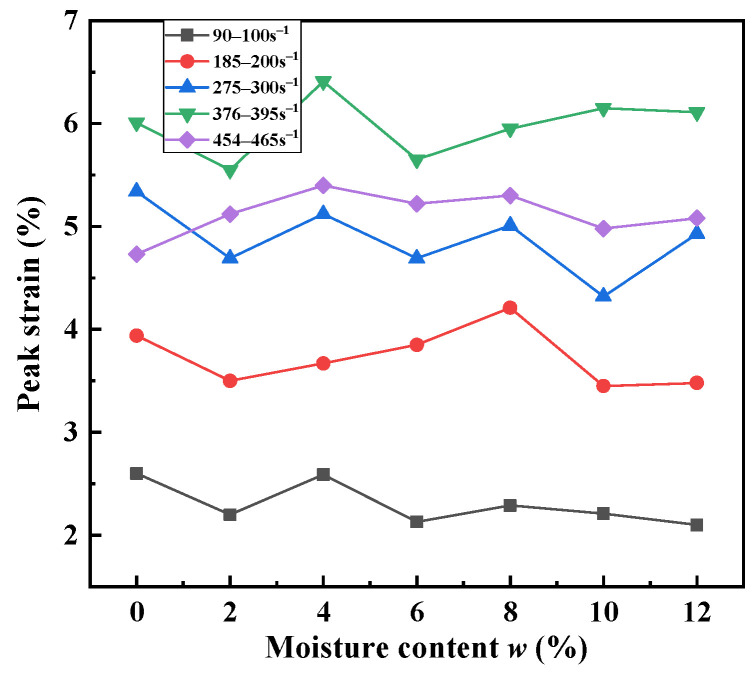
Variations in peak strain with moisture content for sand specimens at different strain rates.

Figure 19 and Figure 20 show that the peak stress and peak strain of the sand specimens changed very complicatedly with the water content. Two transitional moisture content values (8% and 10%) appeared in the current research study. As water was progressively added from 2% to 4% to 8%, the peak stress of the sand specimens fluctuated, and their peak strain slowly increased. When the moisture content exceeded 10%, the peak stress increased, and the peak strain tended to be stable. The sand specimens were the softest when the moisture content was 10%.

This phenomenon was mainly attributed to the interaction mechanism of pore water, pore air and sand particles. The saturation and air volume percentage values corresponding to the moisture contents are given in Table 3. It was found that the percentage of air volume decreased with the increase in the moisture content. For relatively low values of moisture content (*w* < 8%), the main functions hindering sand deformation were the friction and occlusion among particles. It is known that water occupies most of the contact surface of sand particles [49]. The lubricating effect of water reduces the interactions among the particles and the compression resistance. Pore water dissolves a small amount of pore air. Sand particles easily slide relatively to each other. Deformability increases with the water content on a macroscopic scale. However, the dynamic stress of low-saturated sand reported in [32,49,50,51] tended to decrease compared with dry sand. No obvious decreasing trend was observed in the current study, which may have been related to the SHPB instrument used in the experiment. Compared with the bullet, the length of the specimens was too great, resulting in a long time being necessary to obtain the stress balance of the specimens. Luo et al. [9] also reported a slight increase in stress at moisture contents of 4.2% and 8.2%, but the authors considered that the deviation was negligible. Furthermore, the dynamic mechanical behavior of low-moisture-content sand is not sensitive to water for dense sand.

For higher degrees of saturation (*w* > 10%), the pore volume in the sand specimens decreased. More water occupied the space between the pores as the volume of water increased. Pore water dissolved a large amount of pore air when the specimens were subjected to dynamic impact. The saturation of the specimens was increased. The contribution of the water particles to the supporting of the applied load increased. The incompressibility of water made the specimens stiffen. The peak stress of the sand specimens increased with the moisture content, but the deformability decreased. From this point of view, the increase in the moisture content improved the stiffness of the material. It is worth mentioning that when the strain increased enough to completely displace the air in the pores, sand was completely saturated in the test, which then transformed into the loading of a water-and-sand two-phase material. The stiffness of soil increased rapidly as the strain continued to increase. This was also confirmed in [32]. When 8% < *w* < 10%, the sand specimens were in the transition between the above two deformation mechanisms. The influence of moisture content on the dynamic mechanical properties of sand was not significant. In addition, the lubrication effect of water also affected the boundary conditions between the sleeve and the specimens [52]. The resistance between the sleeve and the specimens was reduced, and the specimens were more easily compressed.

### 3.3. Energy Dissipation Analysis

Particle breakage is the process of energy transfer and dissipation. Research on energy dissipation essentially contributes to the understanding of failure characteristics. In the current research study, the incident energy carried by the incident wave was input and converted into reflected energy, absorbed energy and transmitted energy through the SHPB device. Other energy losses were ignored during the test. The absorbed energy, $W_{4}$, of a material is defined as:

(12) $$W_{4}=W_{1}-W_{2}-W_{3}$$

where $W_{1}$, $W_{2}$ and $W_{3}$ are the incident energy, reflected energy and transmitted energy carried by the incident wave, reflected energy and transmitted energy, respectively. They are defined as:

(13) $$W_{i}=\int_{0}^{t}\varepsilon_{i}^{2}\left(t\right)dt,\;i=1,2,3$$

where $\varepsilon_{1}\left(t\right)$, $\varepsilon_{2}\left(t\right)$ and $\varepsilon_{3}\left(t\right)$ are the strains corresponding to the incident wave, reflected wave and transmitted wave, respectively. In order to eliminate the size effect, the specific energy absorption (*SEA*) was adopted to represent the energy dissipation of the specimens, which is defined as the energy absorbed by per unit volume of a specimen. It can be expressed as:

(14) $$SEA=\frac{W_{4}}{V}$$

where V is the volume of the specimen. In the current test study, the acting time of the impact load was very short, and the friction between specimens and sleeve was ignored. The temperature of the specimens remained unchanged during impact compression. Therefore, it was considered that the impact energy was all spent for specimen destruction, and no energy was transformed into heat.

#### 3.3.1. Effect of Initial Relative Density

**Figure 21:**
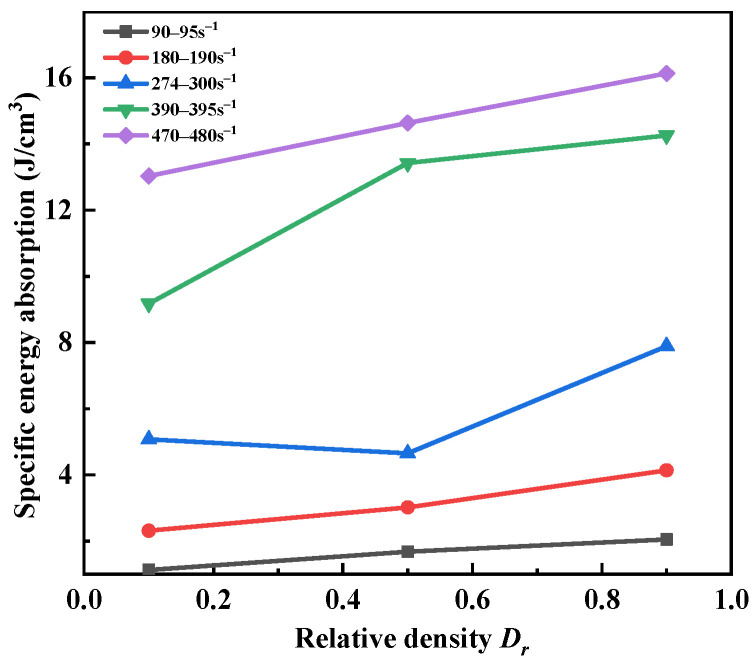
Variations in specific energy absorption with relative density at different strain rates.

The variations in the specific energy absorption with relative density at different strain rates are presented in Figure 21. It was found that the *SEA* of sand specimens generally increased with the initial relative density. Friction resistance was the main mechanism that opposed the deformation and slip of dry particles. The initial pores of the dense sand specimens were small. The intimate arrangement of sand particles resulted in the enhancement of friction and occlusion among the sand particles. This means that denser specimens consumed more energy within a defined volume. On the other hand, the specific absorption energy was very sensitive to the strain rate and obviously increased with the increase in the strain rate. The high strain rate was caused by the high-speed impact, which input a lot of energy into the specimens. The sand specimens absorbed energy, and the deformation increased. However, the increase in the strain rate weakened the enhancement effect of the initial relative density on the SEA. When the initial relative density increased from 0.1 to 0.9, the *SEA* increased by 81.42% when the strain rate was 90~95 s^−1^ but only by 23.79% when the strain rate was 470~480 s^−1^. The particle breakage of the sand specimens was increased at high strain rates, which led to energy dissipation.

#### 3.3.2. Effect of Moisture Content

**Figure 22:**
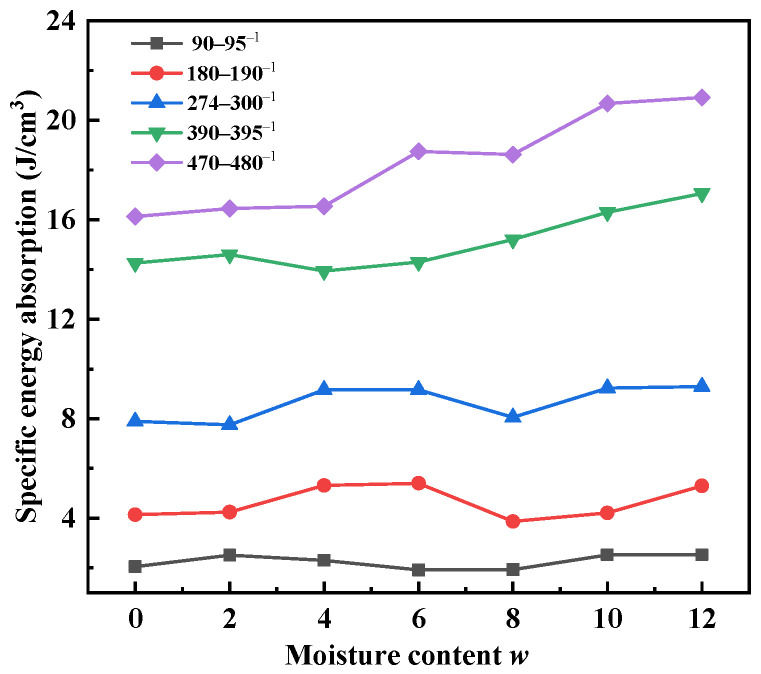
Variations in specific energy absorption with moisture content at different strain rates.

The influence of moisture content on the specific energy absorption is presented in Figure 22. The test results show that the SEA of the sand specimens showed a rising trend with the moisture content, and the rising rate at high strain rates was slightly higher than that at low strain rates. When the moisture content increased from 0 to 12%, the *SEA* increased by 22.9% and 29.7% at strain rates of 90~95 s^−1^ and 470~480 s^−1^, respectively. Pore water softened the sand specimens and reduced the wave impedance. The ability of sand specimens to absorb and dissipate input energy was enhanced. The softening effect was amplified at high strain rates.

## 4. Conclusions

In the current investigation, in order to explore the influence of the initial physical state on the dynamic response of sand, a series of dynamic compressive tests on sand with particle sizes of 0.25–1 mm were carried out. A steel sleeve was designed to ensure the specimens were loaded under the passive confining pressure. The SHPB was modified using pulse shaping techniques. A set of test techniques suitable for reporting the impact dynamic behaviors of sand were proposed and applied. The experimental results were examined from the perspectives of particle breakage, stress–strain relationship and energy absorption. The conclusions of the current study can be summarized as follows:

1. The particle breakage of sand under dynamic compression was sensitive to the strain rate and moisture content. Sand particle breakage increased with the strain rate. Sand specimens had a damage limit when subjected to dynamic impact. With the increase in the moisture content, the relative breakage rate of sand specimens gradually decreased. The presence of moisture lubricated the sand particles, and the degree of lubrication increased with the moisture content;
2. The dynamic response of sand under dynamic compression showed an obvious strain-rate effect. The stress of the specimens increased with the strain rate but decreased when the strain rate exceeded 455 s^−1^;
3. The deformation properties of sand specimens were limited. For unsaturated sand, 8% and 10% were the transitional values of the moisture content. The peak strain showed different trends with moisture content on both sides of the transitional values. As the relative density, *D*_r_, increased, the ability to resist the dynamic compression of sand specimens was enhanced;
4. Energy dissipation was quantitatively analyzed. The specific absorption energy increased with the initial relative density and moisture content. The *SEA* of the sand specimens gradually increased with the increase in the initial relative density. However, the rate of increase decreased with the increase in the strain rate. When the moisture content increased from 0 to 12%, the *SEA* increased by 22.9% and 29.7% at strain rates of 90~95 s^−1^ and 470~480 s^−1^, respectively.

## Figures and Tables

**Table 1:** Physical parameters of sand specimens.

Specific Gravity d_s_	Maximum Density ρ_dmax_ (g/cm^3^)	Minimum Density ρ_dmin_ (g/cm^3^)	Median Size d_50_ (mm)
2.63	1.700	1.538	0.6

**Table 2:** Loading cases of current test study.

Group Number	Moisture Content (%)	Relative Density $D_{r}$	Impact Velocity (m/s)
1	0	0.1	6, 7, 9, 11, 12.5
		0.5	
		0.9	
2	2 4 6 8 10 12	0.9	

**Table 3:** Saturation and percentage of air volume for a given moisture content.

Moisture Content (%)	Saturation (%)	Volume of Air (%)
2	9	33
4	19	29
6	28	26
8	37	23
10	47	19
12	56	16

## Data Availability

The raw/processed data required to reproduce these findings cannot be shared at this time as the data also form part of an ongoing study.
